# Maternal undernutrition results in transcript changes in male offspring that may promote resistance to high fat diet induced weight gain

**DOI:** 10.3389/fendo.2023.1332959

**Published:** 2024-01-17

**Authors:** Tiffany K. Miles, Melody L. Allensworth-James, Angela K. Odle, Ana Rita Silva Moreira, Anessa C. Haney, Alex N. LaGasse, Allen J. Gies, Stephanie D. Byrum, Angelica M. Riojas, Melanie C. MacNicol, Angus M. MacNicol, Gwen V. Childs

**Affiliations:** ^1^ Department of Neurobiology & Developmental Sciences, University of Arkansas for Medical Sciences, Little Rock, AR, United States; ^2^ Department of Radiology, University of Texas Health Science Center at San Antonio, San Antonio, TX, United States

**Keywords:** maternal nutrition, diet induced obesity, developmental programming, IUGR (Intrauterine growth restriction), thrifty phenotype hypothesis, transcriptome (RNA-seq), neonatal leptin surge, metabolism and endocrinology

## Abstract

Maternal nutrition during embryonic development and lactation influences multiple aspects of offspring health. Using mice, this study investigates the effects of maternal caloric restriction (CR) during mid-gestation and lactation on offspring neonatal development and on adult metabolic function when challenged by a high fat diet (HFD). The CR maternal model produced male and female offspring that were significantly smaller, in terms of weight and length, and females had delayed puberty. Adult offspring born to CR dams had a sexually dimorphic response to the high fat diet. Compared to offspring of maternal control dams, adult female, but not male, CR offspring gained more weight in response to high fat diet at 10 weeks. In adipose tissue of male HFD offspring, maternal undernutrition resulted in blunted expression of genes associated with weight gain and increased expression of genes that protect against weight gain. Regardless of maternal nutrition status, HFD male offspring showed increased expression of genes associated with progression toward nonalcoholic fatty liver disease (NAFLD). Furthermore, we observed significant, sexually dimorphic differences in serum TSH. These data reveal tissue- and sex-specific changes in gene and hormone regulation following mild maternal undernutrition, which may offer protection against diet induced weight gain in adult male offspring.

## 1 Introduction

Maternal levels of nutrition during gestation and lactation play a critical role in offspring development and adult metabolic health (1, 2). The earliest studies of maternal undernutrition affecting offspring metabolic function was from the Dutch “Hunger Winter”, in which a short-time famine occurred from 1944-1945. A review of the Dutch “Hunger Winter” cohort study found that in offspring born to mothers exposed to the famine in late gestation weighed less and were shorter, had smaller head circumferences at birth and that the placenta was less efficient (3, 4). These characteristics represent the hallmarks of intrauterine growth restriction (IUGR) (4–6).

The adult offspring of famished mothers were at an increased risk for metabolic syndrome compared to people not exposed to the maternal nutrient restriction (7, 8). Men exposed to the famine during late gestation were less likely to become obese compared to those who were exposed in early gestation (9). Studies have demonstrated that the prenatal environment affects DNA methylation and is dependent on gestational time of exposure and sex (10, 11).

These studies in humans have led to a fundamental hypothesis called the “thrifty phenotype”, initially proposed by Hales and Barker in 1992, and associates poor fetal growth with the development of impaired glucose tolerance and the metabolic syndrome in adulthood (12, 13). Undernutrition in late gestation or during lactation disrupts the neuroendocrine axes and alters the timing of pubertal development, resulting in altered neonatal IGF-1 and GH serum levels and decreased glucose tolerance in adulthood (14). IUGR influences growth, cognitive development, cardiac function, and metabolic homeostasis into adulthood (15, 16).

Researchers are still defining the role of the metabolic signaling adipokine, leptin, in embryonic development and adult health. During the rodent postnatal period and third trimester of humans, there is a leptin surge that occurs independent of adiposity (17). This postnatal leptin surge plays a vital role in the development of the hypothalamic wiring and multiple other organs, including browning of adipose tissue (18–21). The timing and amplitude of the leptin surge was found to be altered by nutrient availability during lactation, through altering litter size (22).

In 2005, the Yura et al. study was the first to demonstrate a premature shift in the neonatal leptin surge as a result of a 30% maternal undernutrition in C57BL/6 mice (23). The undernutrition started in mid-gestation and the neonatal leptin surge was characterized by combining male and female data, since there were no observable sex differences in the leptin surge. Male offspring were only used after weaning and challenged with the 60% high fat diet (HFD) as adults. They had increased weight gain on the 60% HFD and exogenous leptin administered at the peak of the premature surge was shown to replicate the increased HFD induced weight gain, as seen by the maternal undernutrition model. Using the C57BL/6 mouse, this study demonstrated that maternal undernutrition connected the premature shift in the neonatal leptin surge to adult weight gain following HFD exposure.

Our ongoing studies, using leptin receptor (LEPR) knockout “sighted” FVB mice, demonstrate the significance of somatotrope regulation by leptin and the necessity for determining the effect of a neonatal leptin surge on adult pituitary function (24–26). The focus of this study was to use a maternal undernutrition model (a non-genetic method) to shift the offspring neonatal leptin surge and determine the impact on offspring pituitary and metabolic function in adulthood, especially when challenged by nutrient stress (excess caloric fat). In this study, we show that a mild maternal murine undernutrition, starting in late gestation, caused a 3-day advance in the postnatal leptin surge and affected the way in which these offspring responded to a HFD as adults. We report sex differences in responses, including the important finding that males from underfed dams did not gain weight on the HFD, unlike controls (from *ad libitum* fed dams), which differs from what was expected by the “thrifty phenotype” hypothesis and the Yura et al. study (23). RNA-sequencing analysis (RNA-seq) of epididymal white adipose tissue (eWAT), liver and pituitaries from males born to undernourished and *ad libitum* fed dams reveal distinct tissue responses to the HFD and maternal undernutrition. These data offer insight into tissue specific regulation that affords protection from HFD-induced weight gain following a nutrient deficient developmental condition.

## 2 Materials and methods

### 2.1 Animal husbandry

All animal care protocols were approved by the UAMS Institutional Animal Care and Use Committee. FVB.129P2-Pde6b+ Tyrc-ch/AntJ (sighted FVB) mice were housed 1-4 animals/cage at 22.5°C with a 14-hour light (06:00-20:00) and 10-hour dark cycle. Dams for maternal undernutrition breeding were 8-11 weeks of age and bred with 3–4-month-old males. Breeder dams were fed a diet where 20.2% kilocalories (kcal) derived from protein, 21.4% kcal from fat, and 58.4% kcal from carbohydrates (5V5M, PicoLab). At postnatal day (PND) 21, offspring were weaned. Non-breeding mice were fed a diet consisting of 23.086% protein, 14.787% fat, and 62.127% carbohydrates (5V5R, PicoLab).

### 2.2 Establishing the maternal undernutrition model

**Figure 1:**
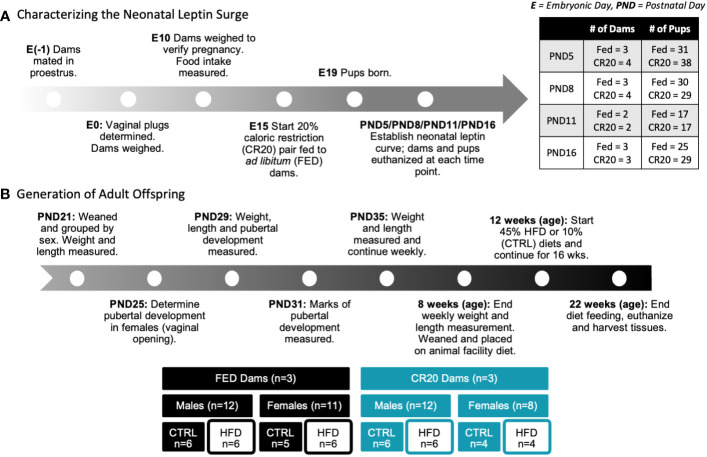
Schematic of experimental design. **(A)** Dam matched feeding, either *ad libitum* (FED) or calorically restricted by 20% compared to *ad libitum* mice (CR20), began at embyronic day 15 (E15). Pups were either euthanized at time points (PND 5, 8, 11 or 16) or continued to weaning. **(B)** Adult offspring were weight matched at weaning (PND21) within treatment groups and administered either the 10% fat diet (CTRL) or 45% high fat diet (HFD) for 10- or 16-weeks.

**Figure 1** summarizes the steps involved with establishing the 20% calorie restriction model. Male FVB.129P2 mice were singly housed for at least 24 hours prior to mating. Female FVB.129P2 mice were introduced to males in the evening of proestrus (embryonic day -1), which was determined by vaginal cytology. Vaginal plugs were visualized the next morning to confirm that mating occurred. Embryonic day 0 (E0) was established as the day after mating, when the vaginal plug was visualized. On E0, mated females were singly housed and weighed. At E10, females were again weighed to verify pregnancy with an expected 3 g increase in weight from E0. Pregnant females, or dams, received ~25 g of food daily starting at E12 to visually acclimate to receiving less food. Dams were paired into two feeding groups: *ad libitum* (FED) and 20% calorie restricted (CR20). At E15, the caloric restriction began, with CR20 dams receiving 80% of the food the paired FED dam consumed from the previous day. Food intake was measured daily around 0930. The undernutrition continued until the offspring were euthanized or weaned. There was one exception to this protocol: The day before parturition, the FED dams consumed less than 3 g of food. To prevent CR20 dams from consuming young at any point in the study, 3 g of food was the minimum made available to CR20 dams. Therefore, if the FED dams consumed less than 3 g of food before parturition, available food to undernourished dams was not reduced below 3 g. Pups were born around E19, with the average litter size being 8 ± 1 pups.

### 2.3 Characterizing FVB neonatal leptin curve induced by CR20 model

The neonatal leptin curve was determined by measuring leptin in the serum of pups at the following time points: PND1, PND5, PND8, PND11, and PND16. To generate a significant number of pups, 3 dams for each nutrient condition (FED or CR20) were used for each time point, except for PND1 where one dam was used for each condition. This resulted in ~28 pups/nutrient condition/time point. At euthanasia, dams and pups were anesthetized with isoflurane (Piramal Critical Care) and decapitated by guillotine (dams) or scissors (pups). Dams were euthanized prior to pups to prevent their stress response caused by pup removal. Trunk serum was collected into ice cold Eppendorf tubes (pups) or conical tubes (dams), and pituitaries collected in 150 μL RIPA (Sigma Aldrich, R0278) containing 10 μL/mL protease inhibitor cocktail (Sigma-Aldrich, P8340). To confirm sex, pup tail snips were collected and genotyped to determine the presence or absence of the male-specific “sex-determining region Y” gene, *Sry*, as previously described (27).

### 2.4 Diet induced obesity of adult CR20 offspring

For the diet induced obesity (DIO) studies, additional offspring were generated using the CR20 model as described above (see 2.2). FED and CR20 offspring were weaned at PND21 and separated by sex. Offspring weight and length (nose to anus) were measured weekly from weaning to 8 weeks of age. Female pups were inspected daily from PND 21 until the day of vaginal opening to determine the timing of puberty.

At 8 weeks of age, pups from each dam were blindly assigned to either a CTRL or HFD feeding group. Offspring were introduced to either a 10% fat (CTRL) diet (Envigo, TD.110675) or a 45% high fat diet (HFD) (Envigo, TD.06415) which they consumed for 10 weeks or 16 weeks. For each sex, this resulted in four experimental groups with 5 ± 1 mice/group: FED control (FED-CTRL); FED high fat diet (FED-HFD) and calorie restricted control (CR20-CTRL) and HFD (CR20-HFD). Food intake, an average of food consumed divided by the number of mice per cage, and individual mouse weights were measured weekly for the duration of the experiment. At euthanasia, trunk serum, pituitaries, eWAT, and liver were collected.

### 2.5 Pituitary, eWAT and liver collection

Pituitaries were collected in RIPA buffer with 10% protease inhibitor on ice and homogenized for 20 sec with a handheld homogenizer. A 20-30 μL aliquot was collected for RNA extraction, and the remaining homogenate was incubated overnight at 4°C. The next morning, the homogenate was centrifuged at 19283 x g for 20 minutes at 4°C. The supernatant was collected and stored at -20°C for later use. The left lateral lobe of the liver and epidydimal white adipose tissue (eWAT) were extracted and flash frozen in liquid nitrogen. For eWAT and liver, a 20-30 mg selection was excised from frozen tissue and homogenized in RIPA buffer with 10% protease inhibitor. RNA was extracted using the Maxwell 16 LEV simplyRNA Tissue Kit (liver samples, Promega, AS1280) or RNAzol RT (eWAT and pituitary samples, Sigma-Aldrich, R4533) and stored at -80°C. Liver samples to be used specifically for RNA-seq were lysed in Zymo DNA/RNA Shield buffer and RNA extracted using a Zymo column-based protocol.

### 2.6 Serum preparation and analyses

The postnatal leptin surge in our FVB mice was initially characterized with serum collected from pups aged postnatal day (PND) 1, 5, 8, 11, and 16. Ahima et al. (17) and results from a pilot study dictated our selection of PND 1, 5, 8, 11, and 16 for our CR20 study (see Methods 2.3). Whole blood was incubated on ice for 1 hour and centrifuged at 3200 x g for 20 minutes at 4°C. Serum was collected on ice and stored at -20°C until use. Serum leptin levels were measured at a 1:10 dilution using the Mouse/Rat Leptin Quantikine ELISA Kit (RNDSystems, MOB00). Serum IGF-1 was measured at a 1:500 dilution using R&D Systems Mouse/Rat IGF-I/IGF-1 Quantikine ELISA Kit (MG100). Serum pituitary hormones, LH, PRL, ACTH, FSH, and TSH, were measured with at 1:2.5 dilution with Milliplex’s MAP Mouse Pituitary Magnetic Bead Panel - Endocrine Multiplex Assay (MPTMAG-49K). Serum adipokines (IL-6, Insulin, MCP-1, PAI-1, Resistin, TNF-α) were measured undiluted with Milliplex’s MAP Mouse Adipokine Magnetic Bead Panel (MADKMAG-71K). Serum GH was measured at 1:2.5 dilution with Milliplex’s MAP Rat Pituitary Magnetic Bead Panel (RPTMAG-86K-01).

### 2.7 RNA sequencing and bioinformatics analysis

RNA extracted from pituitary, liver, and eWAT were submitted for RNAseq. Libraries for RNAseq were prepared using the TruSeq Stranded Total RNA Library Prep Gold kit (Illumina Inc) according to the manufacturer’s protocol. The resulting libraries were sequenced on the NovaSeq; paired-end 2X100. Libraries were prepared with an Illumina TruSeq Stranded mRNA kit using unique dual-indexing. Quality of sequencing for RNA reads were checked using FastQC. The adaptors and low-quality bases (Q < 20) were trimmed to a minimum of 36 base pairs using Trimmomatic (28). STAR was used to align Mus musculus GRCm39.104 reference genome with reads that passed quality control (29). To obtain raw counts, BAM files using Subread’s features Count function were transformed to log2 counts per million (CPM) (30). Genes with a low expression were filtered out and libraries normalized by trimmed mean of M values (31). R package, Limma, using the voom “With Quality Weights” function was used for differential expression and linear modeling (32). We did both Principal Component Analysis (PCA) and cluster analysis as part of the quality control (**Supplementary Figure 6**). Genes with an FDR adjusted p-values < 0.055 and a Log2FC ≥ 0.58 (1.5-fold increase) were considered significant and designated as differentially expressed genes (DEGs). Protocol was modified from previously described work (33).

Qiagen Ingenuity Pathway Analysis was used to generate potential upstream regulators from DEGs using the “Comparison Analysis” feature. Activation z-score, with associated p-value, was used to generate heatmaps of potential upstream regulators.

### 2.8 Quantitative real time polymerase chain reaction

For qRT-PCR verification of RNA-seq findings, complementary DNA was synthesized from pituitary, liver, and eWAT lysate (iScript, Bio-Rad, 170-8890), and qRT-PCR was performed using Power SYBR Green PCR Master Mix (Applied Biosystems, 4367659) and the QuantStudio 12K Flex system (Applied Biosystems, Life Technologies) as previously published (33). qPCR primers are listed in **Table 1**. Expression of each target was normalized to cyclophilin (*Ppia*), and fold-change was quantified using the delta-delta-CT method.

**Table 1:** Primers used for quantitative real time PCR.

Target	Reference Sequence #	IDT Assay ID	Forward Sequence	Reverse Sequence
Ppia	NM_008907.2	Custom Oligo	TGGTCTTTGGGAAGGTGAAAG	TGTCCACAGTCGGAAATGGT
Gh	NM_008117.3	Custom Oligo	CCTCAGCAGGATTTTCACCA	CTTGAGGATCTGCCCAACAC
Ghrhr	NM_001003685.3	Custom Oligo	ACCCGTATCCTCTGCTTGCT	AGGTGTTGTTGGTCCCCTCT
Ghsr	NM_177330.4	Custom Oligo	TCAGGGACCAGAACCACAAA	CCAGCAGAGGATGAAAGCAA
Lhb	NM_008497.2	Custom Oligo	TGTCCTAGCATGGTCCGAGT	AGGAAAGGAGACTATGGGGTCTA
Fshb	NM_008045.2	Custom Oligo	AGTTGATCCAGCTTTGCATCTT	GCCAGGCAATCTTACGGTCT
Scd1		Custom Oligo	CCCTCCTGCAAGCTCTACAC	CCATGGTGTTGGCAATGATA
Slc2a4		Custom Oligo	CAGCGCCTGAGTCTTTTCTT	GGCATTGATAACCCCAATGT
AdipoQ	NM_009605	Mm.PT.58.9719546	GCAGGATTAAGAGGAACAGGAG	TGTCTGTACGATTGTCAGTGG
Cd84	NM_013489	Mm.PT.58.31422111	CTTGTCCTTGTGTCCTTCGT	CATTTATTCCTCAGTGCAGCTTT
Cd68	NM_009853	Mm.PT.58.32698807	CCATGAATGTCCACTGTGCT	CACCTGTCTCTCTCATTTCCTT
Cd44	NM_009851	Mm.PT.58.12084136	CACCATTTCCTGAGACTTGCT	TCTGATTCTTGCCGTCTGC
Il1rn	NM_031167	Mm.PT.58.31759585	GATTCTGAAGGCTTGCATCTTG	TTGGAAGGCAGTGGAAGAC
Il7r	NM_008372	Mm.PT.58.14297778	TGACTTCCATCCACTTCCAAC	GCTTTCGCTATAGTTTTCTGCTT
Tnfaip2	NM009396	Mm.PT.58.5818667	GCCTCCAGCAATCTGATCT	TGCAGAACCTCTACCCCAA
Mmp12	NM_008605	Mm.PT.58.31615472	GCTCCTGCCTCACATCATAC	GGCTTCTCTGCATCTGTGAA
Pde3b	NM_011055	Mm.PT.58.11768018	CAACTCCATTTCCACCTCCA	GTCGTTGCCTTGTATTTCCC
Igf2bp2	NM_183029	Mm.PT.58.13181364	ACCATCCTCTCACTGACATCT	ACACATCAAACAGCTCGCT
Peg3	NM_008817	Mm.PT.58.5628746	TCTTGTCCTCTTTGAGTTCCAC	CAGAGATGATGACAGACGTTCC

IDT, Integrated DNA Technologies, Inc.

### 2.9 Statistics

Statistical analyses of mRNA and pituitary hormone levels were performed on 6 male mice and 5 ± 1 female mice per condition. For most analyses, a Two-Way ANOVA followed by Newman-Keul’s, Bonferroni’s, or Fisher’s Least Significant Difference *post hoc* tests (p<0.05 was considered significant) was used, unless otherwise indicated. Three-way ANOVA followed by Fisher’s LSD test or Student’s *t* test was used when appropriate. All statistical methods are described in the individual figure legends. Power analyses have been published (34).

## 3 Results

### 3.1 Impact of 20% maternal calorie restriction on weight and leptin of dams and progeny

This study, to our knowledge, is the first to characterize a model of maternal caloric restriction and subsequent effects on offspring in the “sighted” FVB mouse strain. Therefore, we undertook a comprehensive characterization of food intake, weight gain, and serum markers to determine the metabolic impact of our design on this strain.

**Figure 2:**
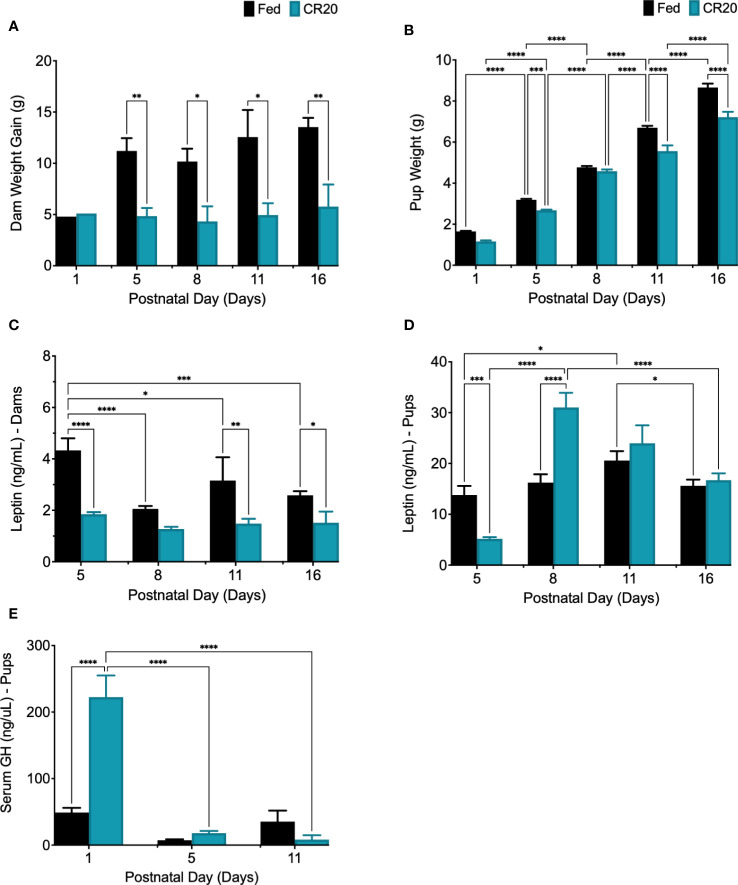
Characterizing postnatal leptin curve in offspring of FED and CR20 dams. **(A)** Dam weight gain (g) from D0 (mating) to euthanasia, PND5 (-6.35g, p = 0.0386) and PND16 (-7.76g, p = 0.0092). **(B)** Pup weight at euthanasia was less in CR20 pups: PND1 (-0.4875g, p<0.0001), PND5 (-0.514g, p = 0.0008), PND11 (-1.141g, p<0.0001), and PND16 (-1.445g, p<0.0001). **(C)** Dam serum leptin. **(D)** Fed pup serum leptin levels peaked at PND11 (+7070pg/mL from PND5 to PND11, p = 0.0260). **(E)** Serum GH was measured in PND1 (+173589pg/mL, p<0.0001), PND5, and PND11 pups by ELISA. For dam, n = 2-4 per condition and time point; error bars are SEM. For pups, n = 17-38 per condition and time point; error bars are SEM. Values that differ significantly between conditions: *p<0.05; **p<0.01, ***p<0.001, ****p<0.0001. Dam Weight: Two-way ANOVA F (4, 17) = 0.9019. Pup Weight: Two-way ANOVA F (4, 202) = 8.913 and Student’s t-test was used for pup weight at PND1. Leptin Dams: Two Way ANOVA F (3, 16) = 4.906. Leptin Pups: Two-Way ANOVA F (3, 130) = 16.40, among FED pup time points: One-way ANOVA F (3, 58) = 3.022, and among CR20 pup time points: One-way ANOVA F (3, 72) = 46.46. Serum GH: Two-way ANOVA F (2, 37) = 19.69; Serum IGF-1: Student’s t test, one-tailed, *p<0.02, t = 2.198, df = 9.

Dam weight gain at PND1 indicated weight gain from E15 to PND1 and showed that FED and caloric restricted (CR20) dams had similar weight gain during pregnancy (**Figure 2A**). Litter size was not significantly decreased by mild maternal undernutrition at each neonatal time point (data not shown). After birth, CR20 dams gained significantly less weight than FED dams at PND5 and PND16 (**Figure 2A**). This lack of weight gain correlated with significantly lower levels of serum leptin in CR20 dams (**Figure 2C**; **Supplementary Figure 1**).

Pups from CR20 dams weighed significantly less than pups from FED dams at PND1, PND5, PND11, and PND16 (**Figure 2B**). Analysis of the postnatal leptin surge revealed that serum leptin peaked at PND11, in both male and female FED pups (**Figure 2D**). Since there were no sex differences for postnatal serum leptin among experimental groups, postnatal male and female data were combined. In pups from CR20 dams, the peak leptin expression was three days earlier (PND8) than that of the FED dam’s offspring (PND11) (**Figure 2D**). Serum leptin levels were significantly lower in CR20 dam pups on PND5 than in FED pups (-9777pg/mL, p<0.0001). On PND8, serum leptin levels from CR20 dam pups elevated by +11656pg/mL (p<0.0001) compared to FED dam pups (**Figure 2D**). This was followed by a decrease at PND11 and PND16 to levels not different from those in FED dam pups (**Figure 2D**). Although serum GH levels were elevated in CR20 dam pups on PND1 (**Figure 2E**), neither postnatal nor adult mice from any group had significant changes in serum GH (Supplementary **Figure 3**). Serum IGF-1 was measured in serum from PND16 mice, but there was no significant difference between FED and CR20 offspring (**Supplementary Figure 1B**).

**Figure 3:**
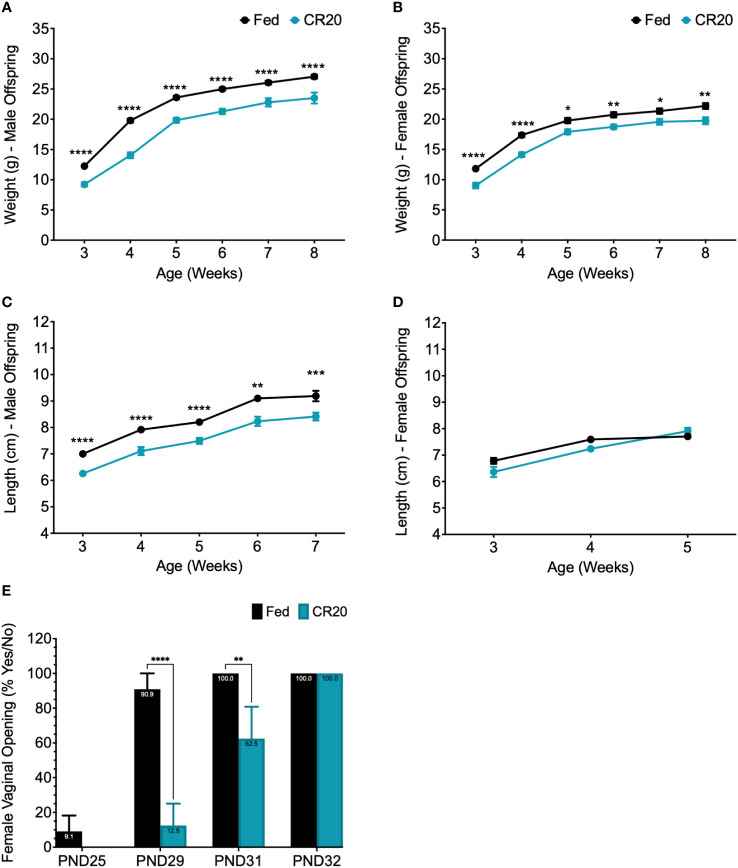
Weight and length of maturing offspring from CR20 and FED dams. The weight (g) of male **(A)** and female **(B)** offspring measured weekly starting at 3 weeks (weaning) to 8 weeks of age. Length (cm), from nose to anus, of male **(C)** and female **(D)** offspring starting at 3 weeks of age. Females were measured until 5 weeks, when length was shown to be not significant among conditions. **(E)** Female vaginal opening was determined (PND25-PND32) and numerical value given for “yes” = 100 and "no" = 0 for each mouse. For male offspring of each condition, n = 12. For female offspring, n = 11 from FED dams and n = 8 from CR20 dams. Values that differ significantly between conditions: *p<0.05; **p<0.01, ***p<0.001, ****p<0.0001. Two-way ANOVA: male weight - F (5, 122) = 3.181, female weight - F (5, 100) = 0.8753, male length - F (4, 86) = 0.09828, and female length - F (2, 51) = 4.641.

### 3.2 Impact of maternal undernutrition on pup maturation

Offspring weight and length were measured weekly from weaning (3 weeks of age) to adult (8 weeks of age). Weight of male and female offspring of CR20 dams increased with age but did not catch up in weight to FED dam offspring (**Figures 3A, B**). This difference in weight was maintained until 8 weeks of age in both sexes (**Figures 3A, B**). Male CR20 offspring were significantly shorter than FED dam pups from weaning to adulthood (**Figure 3C**). In contrast, nose to anus length of female offspring were not significantly different at any time point between CR20 and FED groups (**Figure 3D**). Vaginal opening in the CR20 dam pups was delayed by 2-3 days compared to FED dam pups (**Figure 3E**).

### 3.3 Responses to 10-week high fat diet by progeny from FED and CR20 dams

**Figure 4:**
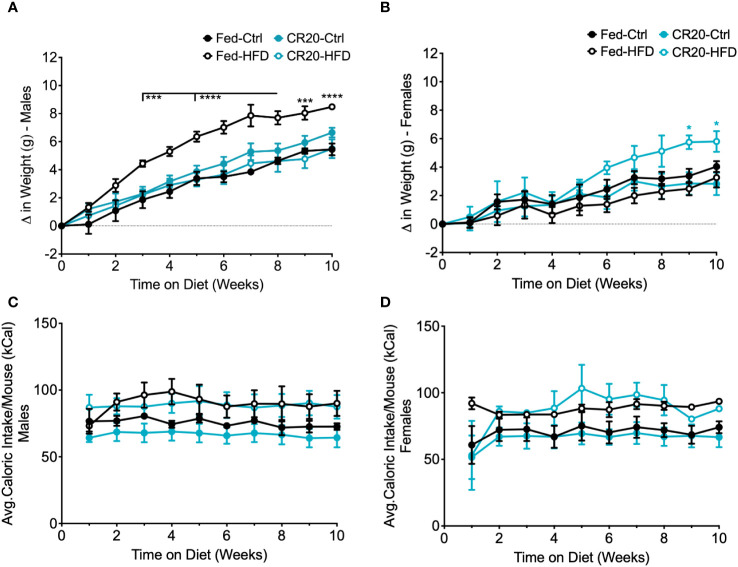
Weekly food intake, weight gain, and serum leptin of CR20 and FED offspring indicate that CR20 male offspring may be protected from diet induced weight gain on 10-week HFD. Weekly difference in weight gain in males **(A)** and female **(B)** due to diet and maternal nutrition. Average caloric food intake (kcal) per mouse for each week on the diet for males **(C)** and females **(D)**. n = 6 per condition for males, n = 5-6 for FED females per condition and n = 4 for CR20 females per condition. Significant difference between HFD vs. CTRL among FED group: ***p<0.001, ****p<0.0001.

The “thrifty phenotype” hypothesis suggests that maternal undernutrition programs offspring for metabolic dysfunction (35, 36) and C57BL/6 male offspring were shown to gain more weight on a high fat diet as adults if born to undernourished dams (23). Our study shows a sex-specific response by underfed progeny to a 10-week (**Figure 4**) treatment of a 45% high fat diet starting at 8 weeks of age.

Among the adult males, only FED dam progeny on the high fat diet (FED-HFD) showed a significant increase in weight (**Figure 4A**). Adult males from the CR20 dams did not gain significantly more weight due to the HFD and thus appeared to be resistant to HFD induced weight gain (**Figure 4A**). In contrast, CR20-HFD females gradually increased in weight starting at 6 weeks on the HFD, and by 8 weeks, weight was significantly different than all three other groups (**Figure 4B**). The average food intake for adult offspring, irrespective of diet, was equivalent in term of kilocalories (Three-way ANOVA, **Figures 4C, D**).

### 3.4 Changes in serum hormone and adipokines in progeny from FED and CR20 dams on 10-week diet

**Figure 5:**
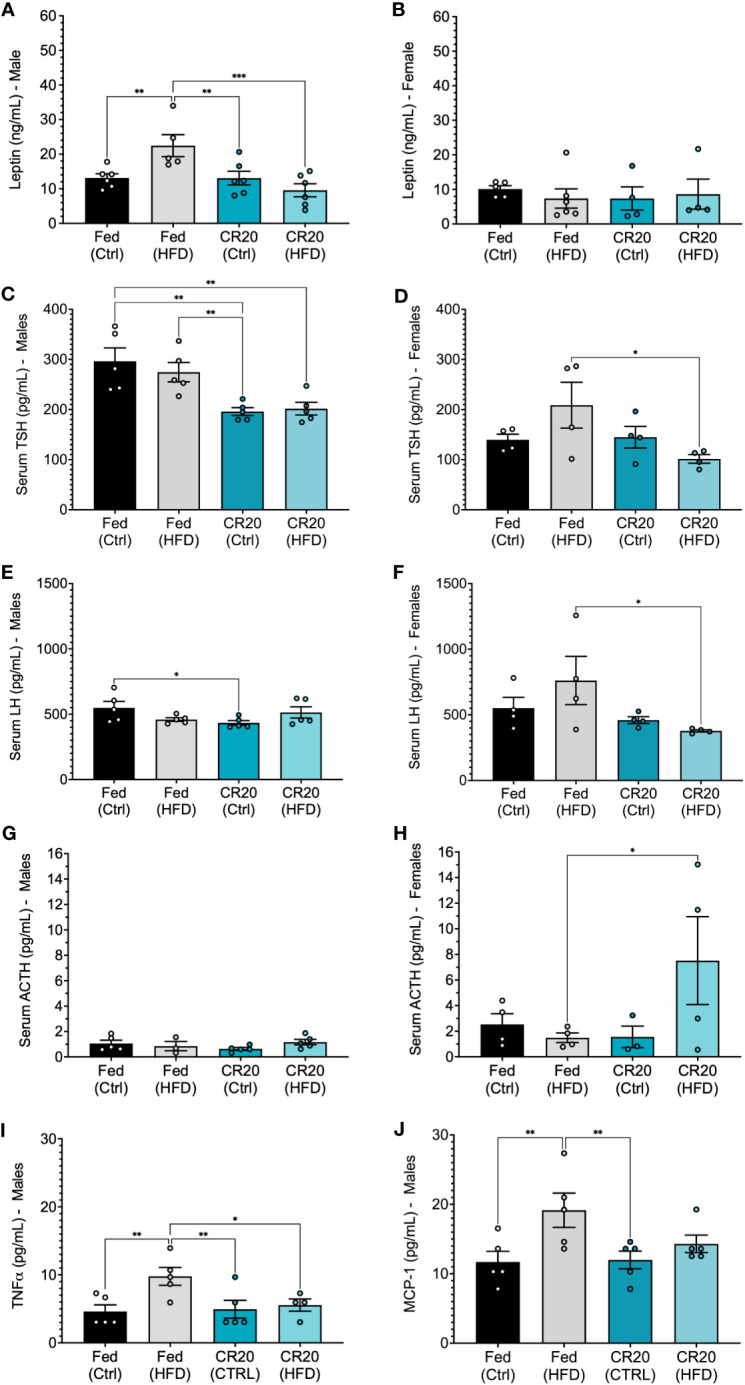
Serum hormone expression in male and female offspring, and male serum adipokines of 10-week treated mice. Leptin levels (ng/mL) in **(A)** male (FED-HFD vs FED-CTRL, +59%, p=0.0365) and **(B)** female CR20 and FED offspring at the conclusion of feeding on the diet. Serum TSH **(C, D)**, LH **(E, F)**, and ACTH **(G, H)** expression in male and female offspring of FED and CR20 dams. Serum adipokines, TNFα **(I)** and MCP-1 **(J)** in male offspring. n = 4-5 per condition for males and n = 4 for females per condition. Error bars are SEM. ANOVA followed by Fisher’s LSD test. *p<0.05; **p<0.01, ***p<0.001.

The adipokine, leptin, is known to correlate with adiposity and assessed to further characterize the effect of maternal diet combined with nutrient stress. We found that correlation with their significant weight gain, FED-HFD males had higher levels of serum leptin compared to FED-CTRL males at 10 weeks of treatment (**Figure 5A**). The lack of weight difference between CTRL and HFD adult males from CR20 dams corresponded with the lack of change in serum leptin (**Figure 5A**). Despite the significant increase in CR20-HFD female weight gain, serum leptin levels were not significantly different among the HFD female groups following 10 weeks (**Figure 5B**).

Since, pituitary products have been shown to be sensitive to nutritional status, we quantified serum hormone changes in the CTRL and HFD adult mice born to either FED or CR20 dams. Serum levels of all major pituitary hormones were quantified in all groups, but only TSH, LH and ACTH were significantly altered (**Figure 5**). TSH is involved in obesity through 1) insulin inhibited deodinase (less T3, high T4), which provides no negative feedback and resulting in high TSH, and 2) leptin is a stimulator of TRH (hypothalamus) which could increase TSH (37). Maternal caloric restriction significantly suppressed TSHß secretion in males regardless of offspring diet (**Figure 5C**). The maternal CR also significantly decreased serum LH levels in CR20-CTRL males (**Figure 5E**) and had no effect on serum ACTH (**Figure 5G**). Adult female offspring showed similar, significant trends in serum levels of both TSHß and LHß; FED-HFD females had slightly *higher* TSHß and LHß than FED-CTRL females, while CR20-HFD females had slightly *lower* serum TSHß and LHß compared to the CR20 controls. This results in statistically significant differences between the FED-HFD and CR20-HFD for both hormones (**Figures 5D, F**). In addition, the female CR20-HFD offspring had significantly increased serum ACTH levels compared to FED-HFD females (**Figure 5H**). No significant differences were found in serum PRL levels across any of the groups.

Since male, but not female, CR20 offspring showed resistance to weight gain in response to the HFD after 10 weeks, male serum was further probed for levels of adipokines and insulin. TNFα and MCP-1 were elevated among FED-HFD males, corresponding with their weight gain and suggestive of adipose tissue inflammation (**Figures 5I, J**). Insulin trended lower in the CR20-HFD males, but this was not statistically significant. The serum adipokines, resistin, IL-6, and PAI-1, were not changed among any of the groups (**Supplementary Figure 3**).

### 3.5 Response by male pituitary, eWAT, and liver transcriptome to maternal undernutrition and 10-week HFD challenge

Though our targeted approach revealed interesting hormonal changes, we next wanted to utilize an unbiased approach to determine the impact of the maternal nutrition-adult nutrient stress model on the global pituitary transcriptome. Since the adult male FED offspring were responsive to the HFD and CR20 offspring showed resistance to weight gain, we focused on males for this unbiased ‘omics’ study. Since, the adipose tissue and liver are central in whole body energy homeostasis, in addition to the pituitary, we included liver and epididymal white adipose tissue (eWAT) in our analyses to discern the genes and pathways involved in the protection against weight gain on a HFD in the CR20-HFD group. All three tissues (pituitary, eWAT, and liver) were assessed by RNA sequencing (RNAseq). Four differential expression comparisons were performed for each tissue’s RNA-seq datasets: CR20-CTRL vs FED-CTRL, CR20-HFD vs FED-HFD, FED-HFD vs FED-CTRL, and CR20-HFD vs CR20-CTRL. Differential expression analysis of the pituitary RNA-sequencing data revealed no significant differences in gene expression among any of the groups with the adj. p-value threshold set at <0.05. (**Supplementary Tables 1**, **2**). This data is supported by modest transcriptomic changes observed by single-cell RNAseq analysis of FVB males exposed to a 60% HFD for 10 and 15 weeks at thermoneutral temperatures (38).

#### 3.5.1 eWAT transcriptomics

**Figure 6:**
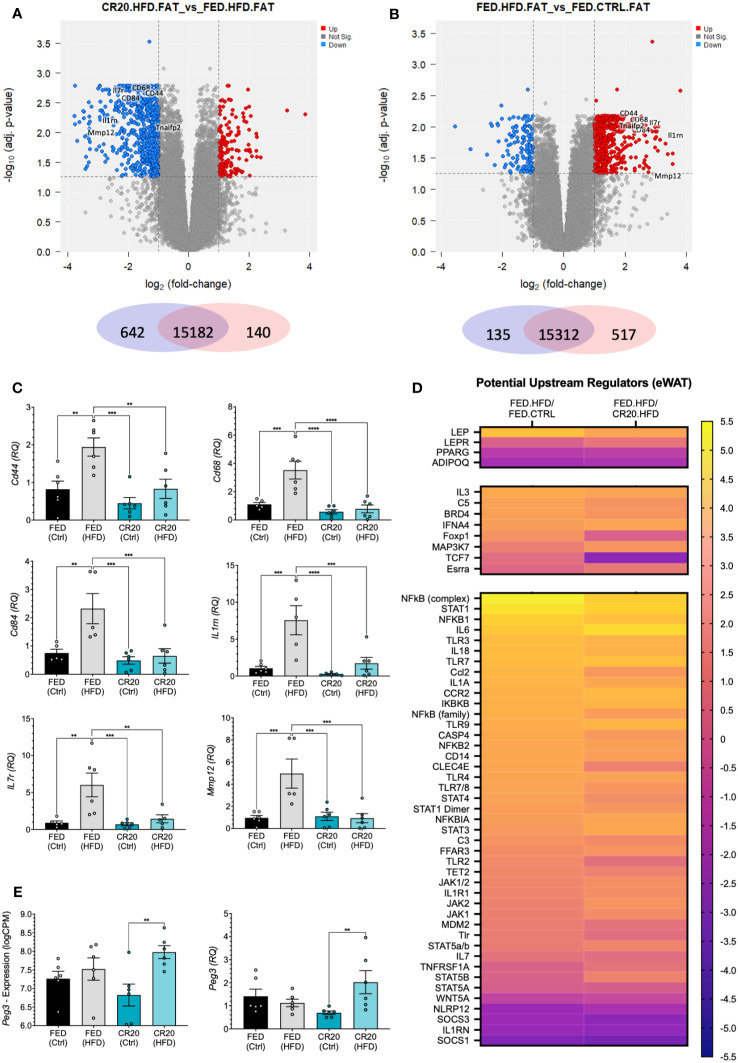
eWAT transcript expression by RNAseq of FED and CR20 male offspring fed either a CTRL or HFD for 10-weeks. Volcano plots of differentially expressed genes (logFC >= 1, adj. p-value <= 0.055) CR20.HFD vs. FED.HFD **(A)** and FED.HFD vs. FED.CTRL **(B)** comparisons. **(C)** qRT-PCR validation of identified cytokines in RNAseq data: *Cd44*, *Cd68*, *Cd84*, *Il1rn*, *Il7r*, and *Mmp12*. **(D)** Heatmap of potentially upstream regulators derived from IPA comparison analysis of DEG’s, separated by genes most associated with obesity, also associated with obesity, and considered protective against obesity. **(E)** Individual mouse RNAseq expression (logCPM, logFC = 1.156, adj. p=0.0018), and qRT-PCR (RQ, FC=2.912, p=0.0064) expression of Peg3. n = 6. Error bars are SEM. ANOVA followed by Fisher’s LSD test. Values that differ significantly between conditions: **p<0.01, ***p<0.001, ****p<0.0001.

Out of the three tissues analyzed, the eWAT had the largest total number of differentially expressed genes. The RNAseq analysis of eWAT revealed statistically significant differentially expressed genes (DEGs) (-log_10_adj. p-value>1.3, log2FC>1) from the following comparisons: CR20-HFD vs FED-HFD (140 genes upregulated, 642 downregulated) and FED-HFD vs FED-CTRL (517 upregulated, 135 downregulated) (**Figures 6A, B**). Notably, there were no significant changes when CR20-HFD eWAT gene expression was compared with that of the CR20-CTRL males (**Supplementary Table 3**). This corresponds well with the lack of weight gain in the CR20-HFD group.

To discern *why* the CR20 males responded differently to the HFD as compared to the FED-HFD, we identified inflammatory cytokines that were differentially expressed in the CR20-HFD vs FED-HFD and FED-HFD vs FED-CTRL comparisons (**Figures 6A, B**; **Supplementary Tables 3**, **4**). Identified cytokines (Cd44, Cd68, Cd84, Il1rn, Il7r, Tnfaip2, and Mmp12) were *upregulated* in the FED-HFD vs FED-CTL but *downregulated* in the CR20 males when both HFD groups were compared. We confirmed these results with qRT-PCR of each target (**Figure 6C**). These same cytokines were *unchanged* in the CR20-HFD compared to CR20-CTRL (**Figure 6C**). Thus, the high fat diet did induce pro-inflammatory gene expression in the eWAT of normal FED dam male offspring, but not in the CR20 male offspring.

The Ingenuity Pathway Analysis (IPA) feature “Comparison Analysis” was used to determine correlations among significantly changed genes (adj. p-value<0.05, logFC>0.58) in the CR20-HFD vs FED-HFD and FED-HFD vs FED-CTRL comparisons. A subsequent upstream regulator analysis inferred the effects that various upstream regulators may be imparting on genes/pathways in our data sets. Predicted upstream regulators effects are designated as either “activated” or “inhibited”, with either a positive or negative activation z-score indicating whether there is increased activation, decreased activation, increased inhibition, or decreased inhibition. Based on existing literature, we identified upstream regulators “associated with obesity”, “closely associated with obesity”, and “protective against obesity when reduced”, all listed in **Supplementary Table 5** (39, 40). Using the z-score we generated a heat map. Initial CR20-HFD vs FED-HFD comparison appeared to directly go the opposite of the FED-HFD vs FED-CTRL regulators. We inverted the analysis, instead comparing FED-HFD vs CR20-HFD, to determine if the CR20-HFD differed from the FED-CTRL. **Figure 6D** illustrates the activation z-score for each of these upstream regulators, and in most cases, the FED-HFD vs CR20-HFD comparison is predicted to change in the same direction as potential upstream regulator with the FED-HFD vs FED-CTRL comparison. This indicates that, when compared to FED-HFD, the CR20-HFD males are not unlike the FED-CTRL males, corresponding with the failure of CR20 males to gain weight on the HFD.

As mentioned, there were no DEGs identified in the CR20-HFD vs CR20-CTRL comparison when our standard constraints were applied (adj. p-value<0.05, logFC>0.58). This is highly significant, as the HFD had seemingly no major effect on gene expression in the eWAT. This is quite the opposite of what was seen with the FED groups, wherein 652 genes were differentially expressed with the HFD treatment. When statistical constraints were relaxed to consider DEGs with a non-adjusted p-value<0.05, we were able to identify a small number of DE genes in the CR20 FED vs HFD comparison (**Figure 6E**). Out of these genes, the most relevant to our study was paternally expressed 3 (*Peg3*), a transcription factor negatively correlated with adiposity (41). In CR20-HFD males, *Peg3* was significantly increased and was the only gene changed with HFD treatment that was specific to the CR20 males. This finding was confirmed with qPCR (**Figure 6E**).

#### 3.5.2 Liver transcriptomics

**Figure 7:**
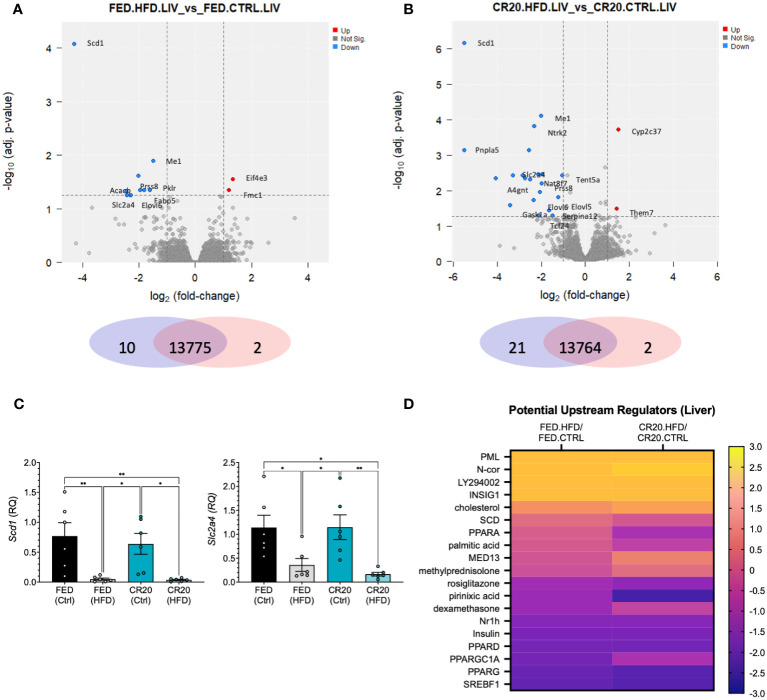
RNAseq liver transcript expression of the same adult male offspring born to FED and CR20 dams that were treated for 10-weeks. Volcano plots of differentially expressed genes (logFC >= 1, adj. p-value <= 0.055) FED.HFD vs FED.CTRL **(A)** and CR20.HFD vs. CR20.CTRL **(B)** comparisons. **(C)** qRT-PCR of *Scd1* and *Slc2a4*, known liver markers of HFD induced obesity, validate RNAseq data. **(D)** Heatmap of potentially upstream regulators derived from IPA comparison analysis of DEG’s associated with NAFLD. n = 6. Error bars are SEM. ANOVA followed by Fisher’s LSD test. Significance designated by: *p<0.05 and **p<0.01.

Maternal undernutrition did not affect how the liver responded to the HFD. RNAseq analysis revealed DEGs (-log_10_adj. p-value>1.3, log2FC>1) only in the FED-HFD vs FED-CTRL (2 upregulated, 10 downregulated) and the CR20-HFD vs CR20-CTRL (2 upregulated, 21 downregulated) comparisons (**Figures 7A, B**; **Supplementary Tables 6**, **7**). *Scd1* and *Scl2a4*, markers of lipid synthesis, were significantly decreased in both HFD groups, as shown by RNAseq and qPCR, which is expected with obesity progression (**Figures 7A–C**) (42, 43). IPA upstream regulator analysis, where HFD was compared to CTRL for each maternal condition, revealed upstream regulators with similar z-scores for the HFD data sets compared to their respective controls (**Figure 7D**; **Supplementary Table 8**). These potential regulators are involved in non-alcoholic liver disease (NAFLD) progression or inhibition (44–47).

### 3.6 Responses to 16-week high fat diet from FED and CR20 progeny

The lack of weight gain by CR20 males differed from the expected increased weight gain suggested by the “thrifty phenotype” hypothesis. We did an initial pilot study to verify findings. More CR20 offspring were generated and subject to a longer treatment time, 16-weeks, on the HFD.

**Figure 8:**
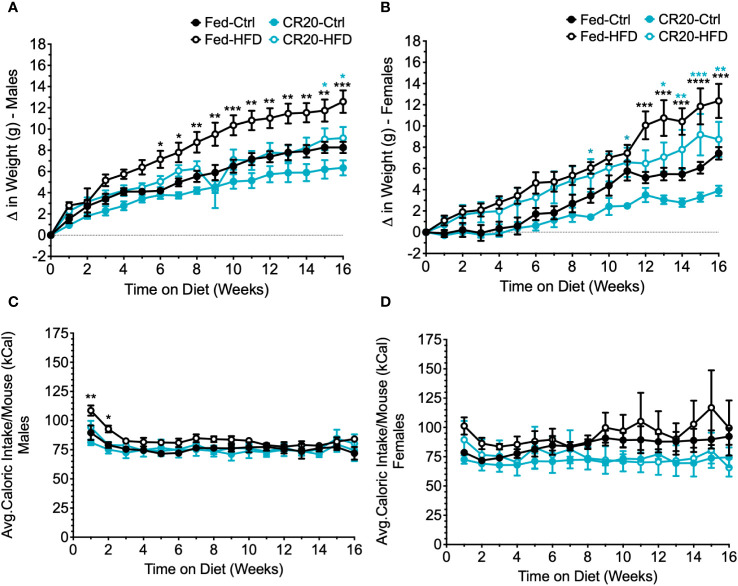
Weekly food intake, weight gain, and serum leptin of CR20 and Fed offspring treated for 16 weeks further indicate that CR20 male offspring are protected from weight gain on 45% fat diet diet and female offspring are partially protected. Average food intake per mouse for each week on the diet for males **(A)** and females **(B)**. Weekly difference in weight from the start of the diet in male **(C)** and female **(D)** offspring. n = 7-8 for males and n = 5-8 for females per condition. Two-Way ANOVA. Values that differ significantly between HFD vs. CTRL of FED group (black asterisks) and HFD vs. CTRL of CR20 group (teal asterisks): *p<0.05, **p<0.01, ***p<0.001, and ****p<0.0001.

Male and female offspring of FED dams showed significant weight gain with the increased treatment time on the high fat diet (**Figures 8A, B**). As observed in the 10-week treatment time, adult males from the CR20 dams did not gain significantly more weight with HFD, appearing again to be resistant to HFD induced weight gain (**Figure 8A**). With the longer treatment time, we were able to observe the FED-HFD females have significant weight gain by 12 weeks on the diet (**Figure 8B**). CR20-HFD females significant weight gain was seen as early as 9 weeks on the diet (**Figure 8B**) and did not rise to the same extent of the FED-HFD females, appearing to be slightly blunted. Again, the average food intake for adult offspring, irrespective of diet, was equivalent in term of kilocalories (**Figures 8C, D**).

**Figure 9:**
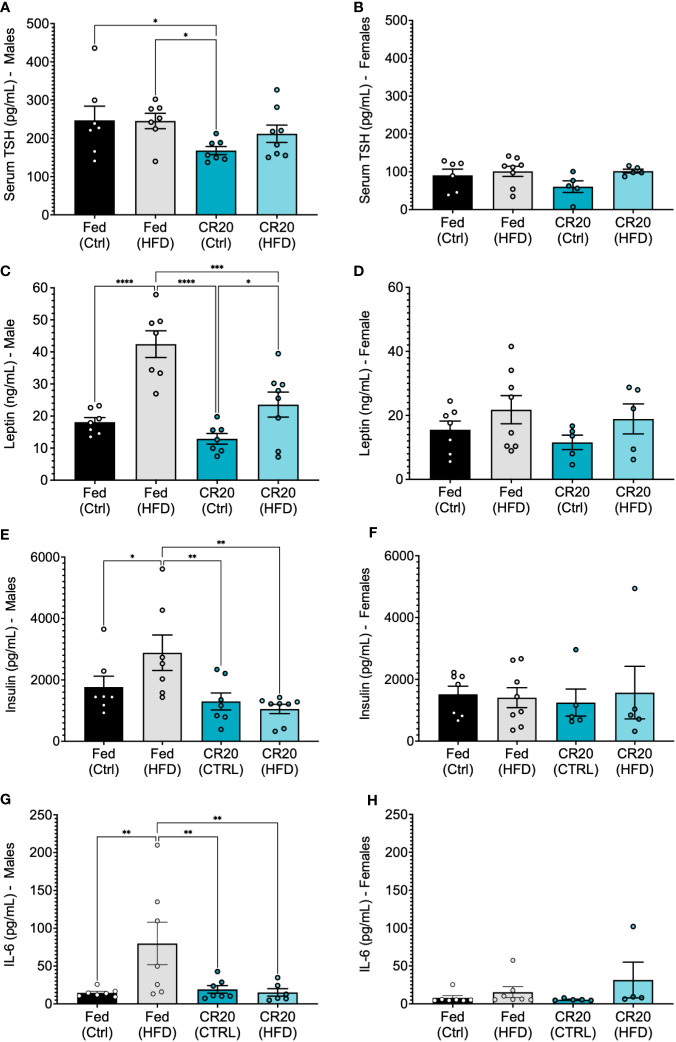
Serum expression of anterior pituitary hormones and adipokines from adult CR20 and FED offspring on the CTRL or HFD for 16-weeks. Serum expression of thyroid stimulating hormone **(A, B)**. Leptin levels (ng/mL) in male **(C)** and female **(D)** CR20 and Fed offspring at the conclusion of diet treatment. Male **(E)** and female **(F)** serum insulin (pg/mL) and IL-6 **(G, H)**. n = 7-8 for males and n = 5-8 for females per condition. ANOVA followed by Fisher’s LSD test. *p<0.05, **p<0.01, ***p<0.001, and ****p<0.0001.

Initial hormone and adipokine analysis of 16-week fed males, supported what was observed with the 10-week feeding. Serum TSH was lower with CR20-CTRL 16-week males (**Figure 9A**), but not significant among females (**Figure 9B**), although trending the same as with the 10-week feeding. Serum leptin was elevated among FED-HFD males, and the longer HFD feeding time caused an increase in serum leptin among the CR20 group, but not to the same extent as the FED (**Figure 9C**). Female serum leptin was not significantly altered but appeared to be shifted in the same direction as with the males (**Figure 9D**). Serum insulin was only elevated in the FED-HFD males at 16-weeks (**Figure 9E**), which was not observed among the 10-week fed group, and insulin was unchanged in females among all conditions (**Figure 9F**). In addition, IL-6 was also only significantly elevated among the FED-HFD males (**Figure 9G**), but not among females (**Figure 9H**).

## 4 Discussion

We have previously characterized the role of leptin signaling to pituitary somatotropes and gonadotropes using selective deletion of *LepR* in sighted FVB mouse lines (24, 26, 34, 48–51). To better allow conclusions across studies and increase knowledge among diverse strains, we wanted to establish a model of maternal undernutrition in sighted FVB mice and investigate the effect of this undernutrition on the physiology of adult offspring challenged by a high fat diet. The mild (20%) caloric restriction model (CR20), starting in late gestation at embryonic day 15 (E15) and continuing through lactation, imparted expected and unexpected consequences upon offspring development and adult health.

### 4.1 Maternal caloric restriction caused delays in growth and pubertal development

“Catch-up growth” refers to an acceleration in growth (measuring body length, weight, organ weight, etc.), possibly due to endocrine or growth plate mechanisms, that permits normal growth progression after early growth retardation (52). The offspring of CR20 dams were born smaller compared to offspring of FED dams, in terms of both length and weight, and the female offspring had delayed pubertal development. These results are consistent with the effects of maternal undernutrition in the literature (53, 54). Decreases in birth length and weight are signs of intrauterine growth restriction (IUGR), a disorder that can be caused by maternal undernutrition (55, 56). Previous studies have also demonstrated that the *timing* of maternal undernutrition during gestation or lactation affects offspring’s propensity for obesity (22, 57). The present study was designed to initiate maternal undernutrition at late gestation and continue through lactation, a paradigm known to cause IUGR and affect the neonatal leptin curve (23, 54). Our CR20-CTRL male mice never showed “catch-up growth” in weight or length and our CR20 females continued to show reduced weight into adulthood.

We were intrigued by our observation of an early surge of leptin in the CR20 dam offspring, suggesting that maternal undernutrition by 20% resulted in an accelerated expression in the offspring of factors that drive adipocytes to secrete the leptin surge. At this point, the identity of the factors that regulate the normal neonatal leptin surge is poorly understood. It is known that the neonatal leptin surge is dependent on maternal nutrient status. In the study presented here, postnatal offspring of undernourished dams had significant alterations in both timing of the leptin surge and levels of leptin throughout the surge period. At PND5, serum leptin levels were lower in CR20 dam offspring than those of FED dam offspring. CR20 dam offspring had an early leptin surge peak, occurring at PND8, which was 3 days earlier than that of the FED dam offspring. However, by the time of the leptin surge peak in the FED dam offspring (PND11), the leptin levels in the CR20 offspring were significantly lower than at PND8 and to the same level as FED PND11.

Our CR20 female offspring had delayed puberty (males were not assessed). Previous studies have shown pubertal delay in both sexes when the neonatal leptin surge was ablated through a 50% maternal undernutrition model in rats (58), decreased hypothalamic regulation of pubertal maturation, and a delay in puberty when the neonatal leptin surge was blocked with a leptin antagonist (59, 60), indicating that pre-pubertal leptin levels can affect the timing of puberty onset in rodents.

### 4.2 Maternal caloric restriction resulted in a premature neonatal leptin surge and male offspring that did not show a high fat diet induced weight gain

In our CR20 model, offspring have a shifted leptin curve, with a peak at PND8, whereas the offspring from FED dams peaked at PND11. A shift in the peak of the leptin surge has been previously shown to have an impact on adult metabolism. Yura et al. (2005) demonstrated that a 30% maternal caloric restriction model resulted in a premature leptin surge which was associated with hypothalamic changes that rendered the animals less sensitive to the anorexigenic effects of leptin (23). The male mice gained more weight on a HFD, supporting the idea that the timing of the leptin surge is important to adult metabolic health. Further examination of the “thrifty phenotype” hypothesis suggests that since fetal development is sensitive to the nutritional environment, the resulting adaptations should improve fetal survival but in the long run may result in metabolic dysfunction in adulthood (52).

Our mild maternal nutrition model reveals responses to maternal undernutrition +/- HFD in adult offspring that are sex-specific. Weight gain to a HFD by female mice born to calorically restricted dams is lacking and could be due to the extensive time (27 weeks) needed to report weight response to the HFD (61). The novelty of this study is that changes in weight, or lack thereof, due to a HFD are reported in “sighted” FVB male and female offspring born to calorically restricted dams. Females born to FED dams did not gain significant weight on the HFD at 10 weeks, while the males did. We reasoned that FVB females on the 45% HFD used in our study would gain excessive weight with a longer treatment time, and our pilot study of the prolonged (16-week) HFD exposure supports this. The female FED-HFD had consistent, significant weight gain starting at 12 weeks, with a final delta weight of 12.4 g. In our studies of the somatotrope *Lepr*-null model, weight gain for females was slower than that of males and not significantly different from control females until 6 months of age (26). We did, however, observe increased adiposity in somatotrope *Lepr-*null females as young as 3-4 months of age (27). We did not assess adiposity in the current study; however, based on experience with the *Lepr*-null model, we conjecture that adiposity may increase before the onset of significant weight gain in FED-HFD females. Ongoing studies will address this question.

The CR20-HFD females gained significant weight earlier (10 weeks) than FED-HFD females. This finding supports studies where maternal undernutrition produced offspring that gained more weight or were more metabolically dysfunctional compared to control diet fed dam offspring (23) and supports the “thrifty phenotype” hypothesis. Thus, the maternal undernutrition may alter metabolic responses in females, so they become more sensitive to a HFD than their FED-HFD counterparts.

However, in sharp contrast, the FED-HFD males, but not the CR20-HFD males, gained significantly more weight, which was detected as early as 3 weeks on the diet. Unexpectedly, male CR20 dam offspring, on either diet, did not gain more weight than FED-FED males at 10 weeks. This indicates that the shift in the leptin surge did not compromise their responses to orexigenic or anorexigenic hormones. Indeed, unlike the FED-HFD males, the CR20-HFD males appeared *resistant* to the HFD. This differs from the Yura et al. (23) findings and could be due to 1) strain difference, C57BL/6 vs “sighted” FVB mouse and 2) start time of the maternal caloric restrictions, embryonic day 10.5 compared to e15 used in this study. Serum leptin was increased with HFD feeding in FED-HFD males, as expected with increased adiposity and weight gain seen in this group. However, leptin levels appeared normal in all other groups, which corresponded with the absence of significant weight gain.

In the serum, we observed significant yet distinct changes in TSH and LH with both sexes. As serum TSH is involved in regulating adipose tissue and found to be high in obese mice (62, 63), reduced serum TSH in male CR20 offspring indicates a TSH mediated mechanism involved in reduced adipose tissue accumulation. Female CR20 on the HFD had reduced TSH levels compared to FED-HFD, which may benefit long term adipose accumulation. Serum LH was reduced in the CR20 groups compared to control counterpart by both sexes, which may indicate prolonged reproductive function that was initially indicated by delayed puberty (64, 65). Elevated female CR20-HFD ACTH may be one of the comorbidities of obesity and is reflected by the advanced weight gain of this group (66, 67). Elevated TNFα and MCP-1, contributors to metabolic disease progression (68, 69), classify the increased weight gain in FED-HFD males as an obese state. We did not observe changes in offspring serum GH, therefore, we believe that ghrelin secretion was not altered due to maternal undernutrition.

### 4.3 Males born to undernourished dams had tissue specific responses to the high fat diet

We observed that weight gain of CR20-HFD males was blunted weight with 10-week of HFD treatment. Therefore, we wanted to determine tissue specific mechanisms that may contribute to this observation. In epidydimal white adipose tissue, significant transcript changes were found with HFD among the FED group and not the CR20 offspring, which corresponded with observed phenotype. Lack of increased inflammatory cytokines by the CR20-HFD mice supported the lack of progression toward obesity and metabolic disease that was seen in the FED-HFD group. Upstream regulator analysis of obesity associated genes and genes protective against obesity illustrate that the adipose tissue of FED-HFD males is poised for obesity development, while CR20-HFD males lack the changes in the genes that promote disease formation and thus are similar to the HFD-CTRL group. For example, the prevalence of *Brd4* promotes obesity associated inflammation (70) and overexpression of proinflammatory cytokines, such as interferons (IFNA4), are associated with obesity pathogenesis (71), which are both elevated only in the FED group. Foxp1-deficient mice were reported to augment energy expenditure and be protected from diet-induced obesity (72), which is predicted to be slightly reduced in CR20-HFD males compared to the FED-HFD males. TCF7, a transcription factor involved in the canonical WNT/β-catenin pathway, was reported to regulate promoter activation of *Ucp1* and *Gprc6a* in brown adipose tissue independent of the Wnt/β-Catenin Pathway (73). TCF7 is proposed to be reduced in CR20-HFD males RNAseq data, which suggests that the lack of weight gain on the HFD may not be due to eWAT browning. TLR3 and TLR7 KOs were found to be protective against HFD induced obesity and weight gain and IL1a-KO reduces adiposity, which reflects predicted gene changes in CR20-HFD males compared to FED-HFD (39).

The transcription factor *Peg3* is a unique gene, as it was specifically changed in the CR20-HFD males, according to p-value, and validated by qRT-PCR. It is reported that *Peg3+/−* mutants develop excess adiposity, despite low food intake, have delays in pubertal development, and are leptin resistant (41). Peg3 is strongly expressed in the hypothalamus and pituitary during development (41). *Peg3* is primarily expressed by the paternal allele due to repression of the maternal allele by DNA methylation. When the imprinting control region was deleted on the maternal allele, transcriptional regulation of *Peg3* paternal allele was upregulated and resulted in increased body weight (74). Prolonged caloric restriction has been shown to alter DNA methylation and increase healthy lifespan (75). Therefore, epigenetic regulation of *Peg3* maternal allele could potentially alter the action of *Peg3*. Further studies are needed to determine if nutrient stress alters the epigenetic regulation of *Peg3* that would alter changes in weight.

Interestingly, the liver of HFD mice, regardless of maternal nutrition, responded equally to nutrient stress and showed indications of NAFLD progression. The liver and adipose tissue form a metabolic axis involving crosstalk, maintenance of energy homeostasis and having essential roles in nutrient uptake, processing, and storage (76–78). “Adipose tissue failure” is a term used to describe the failure of lipid storage and mobility by adipose tissue that results in lipid overload to the liver (79). Although lean, these individuals have NALFD progression but thought to be “metabolically unhealthy lean”, which is approximately 20% of the normal weight population and can have a three-fold greater risk for mortality (80). This observation in humans may account for the dysregulated lipid progression in the liver and eWAT of the CR20-HFD males, and if so, then a mild maternal undernutrition may be involved in metabolically unhealthy progression.

## 5 Conclusion

This study reports that a mild 20% maternal undernutrition in late gestation of mice affects the timing of the offspring neonatal leptin surge so that it peaks prematurely. The timing of this peak is like that reported by Yura et al. (2005) who used a 30% undernutrition model and reported that the adult progeny gained more weight on a HFD than their counterparts born to Fed dams (23). Female progeny of the 20% calorically restricted dams showed hallmarks of the “thrifty phenotype” hypothesis, with being smaller in size, having a delay in pubertal development, and gaining weight on the HFD as adults, but not more so than the control group. In contrast, male progeny from our 20% maternal undernutrition model had reduced weight throughout adult life and were resistant to the 45% HFD induced weight gain yet may have been metabolically unhealthy due to indications of NAFLD progression. Thus, this model shows distinct sex differences in developmental programming. In females, a premature leptin surge may have programmed the hypothalamus and other sensitive organs to become more sensitive to obesogenic regulators, while in males, this premature leptin surge may have conferred protection against adiposity at the expense of liver lipid toxicity.

## Data availability statement

Original datasets are available in the Gene Expression Omnibus under accession number GSE247985. The original contributions presented in the study are publicly available. This data can be found here: https://urldefense.com/v3/__https://www.ncbi.nlm.nih.gov/geo/query/acc.cgi?acc=GSE247985__;!!LFqOYw!pMmVRDxBAtsYN2f-zzT0NrwKrbz4T0JYw1GWHmxOWnhYLcQna-4A0xBnHmhUzwQR1YhyZIIBixVZNgehkiSb$.

## Ethics statement

The animal study was approved by UAMS Institutional Animal Care and Use Committee. The study was conducted in accordance with the local legislation and institutional requirements.

## Author contributions

TM: Conceptualization, Data curation, Formal analysis, Investigation, Methodology, Project administration, Visualization, Writing – original draft, Writing – review & editing. MA-J: Conceptualization, Data curation, Formal analysis, Investigation, Methodology, Project administration, Writing – review & editing, Validation. AO: Conceptualization, Writing – review & editing. AM: Investigation, Writing – review & editing. AH: Investigation, Writing – review & editing. AL: Investigation, Writing – review & editing. AG: Data curation, Formal analysis, Software, Writing – review & editing. SB: Data curation, Formal analysis, Software, Writing – review & editing. AR: Conceptualization, Methodology, Writing – review & editing. MM: Conceptualization, Funding acquisition, Resources, Writing – review & editing. AM: Conceptualization, Funding acquisition, Resources, Writing – review & editing. GC: Conceptualization, Funding acquisition, Resources, Supervision, Writing – review & editing.

## Supplementary material

Supplementary Figure 1 Dam correlation analysis of leptin to weight and serum IGF-1 in PND16 pups. **(A)** Pearson correlation analysis (two-tailed) of weight to leptin for dams revealed a moderate positive correlation for the FED (r=0.6091, p=0.0467) and CR20 (r=0.6794, p=0.0075) groups. **(B)** Serum IGF1 protein levels in PND16 pups was quantified by ELISA. Student’s t test.

Supplementary Figure 2 Pituitary mRNA expression by qRT-PCR of Gh, Ghrhr, and Ghsr in adult CR20 and FED offspring on the Ctrl or HFD for 10-weeks. **(A, B)** Gh, **(C, D)** Ghrhr, and **(E, F)** Ghsr expression in male and female offspring of Fed and CR20 dams. n = 6 per condition for males, n = 5-6 for FED females per condition and n = 4 for CR20 females per condition. Error bars are SEM. ANOVA followed by Fisher's LSD test: *p<0.05.

Supplementary Figure 3 Serum protein expression of GH and adipokines from adult CR20 and FED offspring on the Ctrl or HFD for 16-weeks. GH serum expression in males **(A)** and females **(B)**. The adipokines resistin **(C)**, IL-6 **(D)**, and PAI-1 **(E)** in male offspring of Fed and CR20 dams. n = 7-8 for males and n = 5-8 for females per condition. ANOVA followed by Fisher's LSD test.

Supplementary Figure 4 Pituitary mRNA expression by qRT-PCR of Gh, Lh and Fshb in 16-week treated mice. Pituitary transcript expression of **(A, B)** Gh, **(C, D)** Lh, and **(E, F)** Fshb in male and female offspring of Fed and CR20 dams. n = 4-7 for males and n = 3-4 for females per condition. Error bars are SEM. ANOVA followed by Fisher's LSD test. *p<0.05; **p<0.01, ***p<0.001, ****p<0.0001.

Supplementary Figure 5 Serum protein expression of anterior pituitary hormones and adipokines from adult CR20 and FED offspring on the Ctrl or HFD for 16-weeks. Serum expression of **(A, B)** LH, **(C, D)** ACTH, **(E, F)** PAI-1, and resistin **(G, H)** in male and female offspring of Fed and CR20 dams. n = 7-8 for males and n = 5-8 for females per condition. ANOVA followed by Fisher's LSD test. *p<0.05.

Supplementary Figure 6 PCA plots and clustered dendrograms to evaluate overall correlation structure of RNAseq data. PCA plot and dendogram for pituitary **(A, B)**, eWAT **(C, D)**, and liver **(E, F)**.

Supplementary Table 1 Pituitary RNAseq data of males on 10-week diet. Gene list, log2 counts-per-millions for each sample and comparison analysis of HFD vs. CTRL for Fed and CR20 offspring.

Supplementary Table 2 Pituitary RNAseq data of males on 10-week diet. Gene list, log2 counts-per-millions for each sample and HFD vs. HFD and CTRL vs. CTRL comparison analysis for Fed and CR20 offspring.

Supplementary Table 3 eWAT RNAseq gene list of 10-week treated males, log2 counts-per-millions for each sample and comparison analysis of HFD vs. CTRL for Fed and CR20 offspring, and FED.HFD vs. CR20.HFD comparison.

Supplementary Table 4 eWAT RNAseq gene list of 10-week treated males, log2 counts-per-millions for each sample and HFD vs HFD and CTRL vs CTRL comparison analysis for Fed and CR20 offspring.

Supplementary Table 5 eWAT upstream regulators derived from 10-week treated males RNAseq data set using the Ingenuity Pathway Analysis Comparison Analysis feature that are associated with fat accumulation and protection.

Supplementary Table 6 Liver RNAseq data of 10-week treated males. Gene list, log2 counts-per-millions for each sample and comparison analysis of HFD vs. CTRL for Fed and CR20 offspring.

Supplementary Table 7 Liver RNAseq data of 10-week treated males. Gene list, log2 counts-per-millions for each sample and HFD vs. HFD and CTRL vs. CTRL comparison analysis for Fed and CR20 offspring.

Supplementary Table 8 Liver upstream regulators derived from 10-week treated males RNAseq data set using the Ingenuity Pathway Analysis Comparison Analysis feature that are associated with NAFLD.
