# Supplementary material for: Maternal undernutrition results in transcript changes in male offspring that may promote resistance to high fat diet induced weight gain
Source: Front Endocrinol (Lausanne). 2024 Jan 17;14:1332959. doi: 10.3389/fendo.2023.1332959 (PMC11077627; doi:10.3389/fendo.2023.1332959)
Supplement: Supplementary Figure 1 — Dam correlation analysis of leptin to weight and serum IGF-1 in PND16 pups. (A) Pearson correlation analysis (two-tailed) of weight to leptin for dams revealed a moderate positive correlation for the FED (r=0.6091, p=0.0467) and CR20 (r=0.6794, p=0.0075) groups. (B) Serum IGF1 protein levels in PND16 pups was quantified by ELISA. Student’s t test. [file DataSheet_1.zip › Supplementary Material/Supplementary Material.DOCX]

Supplementary Material

Maternal Undernutrition Results in Transcript Changes in Male Offspring that May Promote Resistance to High Fat Diet Induced Weight Gain

Tiffany K. Miles*, Melody L. Allensworth-James, Angela K. Odle, Ana Rita Silva Moreira, Anessa C. Haney, Alexandra N. Lagasse, Allen J. Gies, Stephanie D. Byrum, Angelica M. Riojas, Melanie C. MacNicol, Angus M. MacNicol, Gwen V. Childs

***Correspondence:** Tiffany K. Miles: [tkmiles@uams.edu](mailto:tkmiles@uams.edu)

Supplementary Figure 1. Dam correlation analysis of leptin to weight and serum IGF-1 in PND16 pups. A) Pearson correlation analysis (two-tailed) of weight to leptin for dams revealed a moderate positive correlation for the FED (r=0.6091, p=0.0467) and CR20 (r=0.6794, p=0.0075) groups. B) Serum IGF1 protein levels in PND16 pups was quantified by ELISA. Student’s *t* test.

Supplementary Figure 2. Pituitary mRNA expression by qRT-PCR of *Gh*, *Ghrhr*, and *Ghsr* in adult CR20 and FED offspring on the Ctrl or HFD for 10-weeks. (A-B) *Gh*, (C-D) *Ghrhr*, and (E-F) *Ghsr* expression in male and female offspring of Fed and CR20 dams. n = 6 per condition for males, n = 5-6 for FED females per condition and n = 4 for CR20 females per condition. Error bars are SEM. ANOVA followed by Fisher's LSD test: *p<0.05.

Supplementary Figure 3. Serum protein expression of GH and adipokines from adult CR20 and FED offspring on the Ctrl or HFD for 16-weeks. GH serum expression in males (A) and females (B). The adipokines resistin (C), IL-6 (D), and PAI-1 (E) in male offspring of Fed and CR20 dams. n = 7-8 for males and n = 5-8 for females per condition. ANOVA followed by Fisher's LSD test.

Supplementary Figure 4. Pituitary mRNA expression by qRT-PCR of *Gh*, *Lh* and *Fshβ* in 16-week treated mice. Pituitary transcript expression of (A-B) *Gh*, (C-D) *Lh*, and (E-F) *Fshβ* in male and female offspring of Fed and CR20 dams. n = 4-7 for males and n = 3-4 for females per condition. Error bars are SEM. ANOVA followed by Fisher's LSD test. *p<0.05; **p<0.01, ***p<0.001, ****p<0.0001.

Supplementary Figure 5. Serum protein expression of anterior pituitary hormones and adipokines from adult CR20 and FED offspring on the Ctrl or HFD for 16-weeks. Serum expression of (A-B) LH, (C-D) ACTH, (E-F) PAI-1, and resistin (G-H) in male and female offspring of Fed and CR20 dams. n = 7-8 for males and n = 5-8 for females per condition. ANOVA followed by Fisher's LSD test. *p<0.05.

**Supplementary Figure 6. PCA plots and clustered dendrograms to evaluate overall correlation structure of RNAseq data.** PCA plot and dendogram for pituitary (A, B), eWAT (C, D), and liver (E, F).

Supplementary Table 1. Pituitary RNAseq data of males on 10-week diet. Gene list, log2 counts-per-millions for each sample and comparison analysis of HFDvsCTRL for Fed and CR20 offspring.

Supplementary Table 2. Pituitary RNAseq data of males on 10-week diet. Gene list, log2 counts-per-millions for each sample and HFDvsHFD and CTRLvsCTRL comparison analysis for Fed and CR20 offspring.

**Supplementary Table 3.** eWAT RNAseq gene list of 10-week treated males, log2 counts-per-millions for each sample and comparison analysis of HFDvsCTRL for Fed and CR20 offspring, and FED.HFDvsCR20.HFD comparison.

Supplementary Table 4. eWAT RNAseq gene list of 10-week treated males, log2 counts-per-millions for each sample and HFDvsHFD and CTRLvsCTRL comparison analysis for Fed and CR20 offspring.

**Supplementary Table 5.** eWAT upstream regulators derived from 10-week treated males RNAseq data set using the Ingenuity Pathway Analysis Comparison Analysis feature that are associated with fat accumulation and protection.

**Supplementary Table 6.** Liver RNAseq data of 10-week treated males. Gene list, log2 counts-per-millions for each sample and comparison analysis of HFDvsCTRL for Fed and CR20 offspring.

**Supplementary Table 7.** Liver RNAseq data of 10-week treated males. Gene list, log2 counts-per-millions for each sample and HFDvsHFD and CTRLvsCTRL comparison analysis for Fed and CR20 offspring.

**Supplementary Table 8.** Liver upstream regulators derived from 10-week treated males RNAseq data set using the Ingenuity Pathway Analysis Comparison Analysis feature that are associated with NAFLD.
