# Supplementary figures and images for: Maternal undernutrition results in transcript changes in male offspring that may promote resistance to high fat diet induced weight gain
Source: Front Endocrinol (Lausanne). 2024 Jan 17;14:1332959. doi: 10.3389/fendo.2023.1332959 (PMC11077627; doi:10.3389/fendo.2023.1332959)

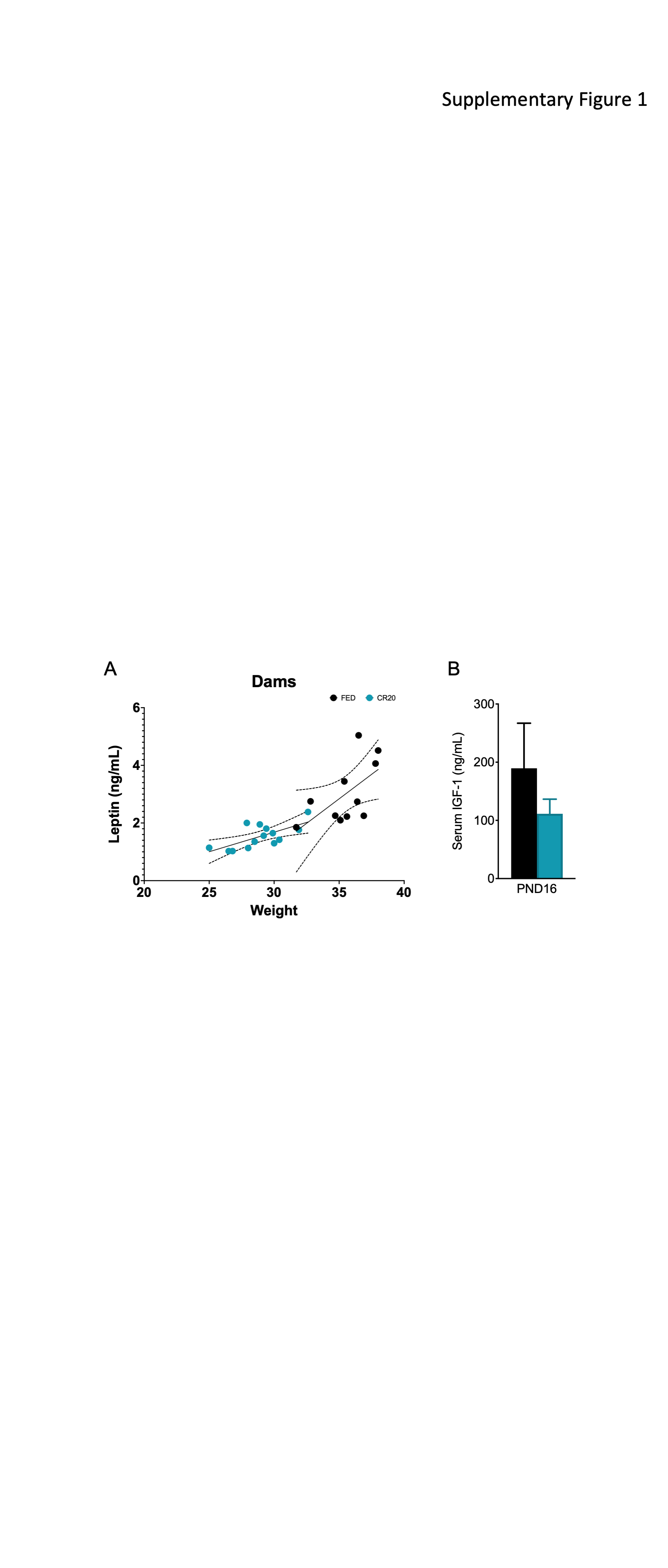

Supplement: Supplementary Figure 1 — Dam correlation analysis of leptin to weight and serum IGF-1 in PND16 pups. (A) Pearson correlation analysis (two-tailed) of weight to leptin for dams revealed a moderate positive correlation for the FED (r=0.6091, p=0.0467) and CR20 (r=0.6794, p=0.0075) groups. (B) Serum IGF1 protein levels in PND16 pups was quantified by ELISA. Student’s t test. [file DataSheet_1.zip › Supplementary Material/Supplementary Figure 1.TIFF]

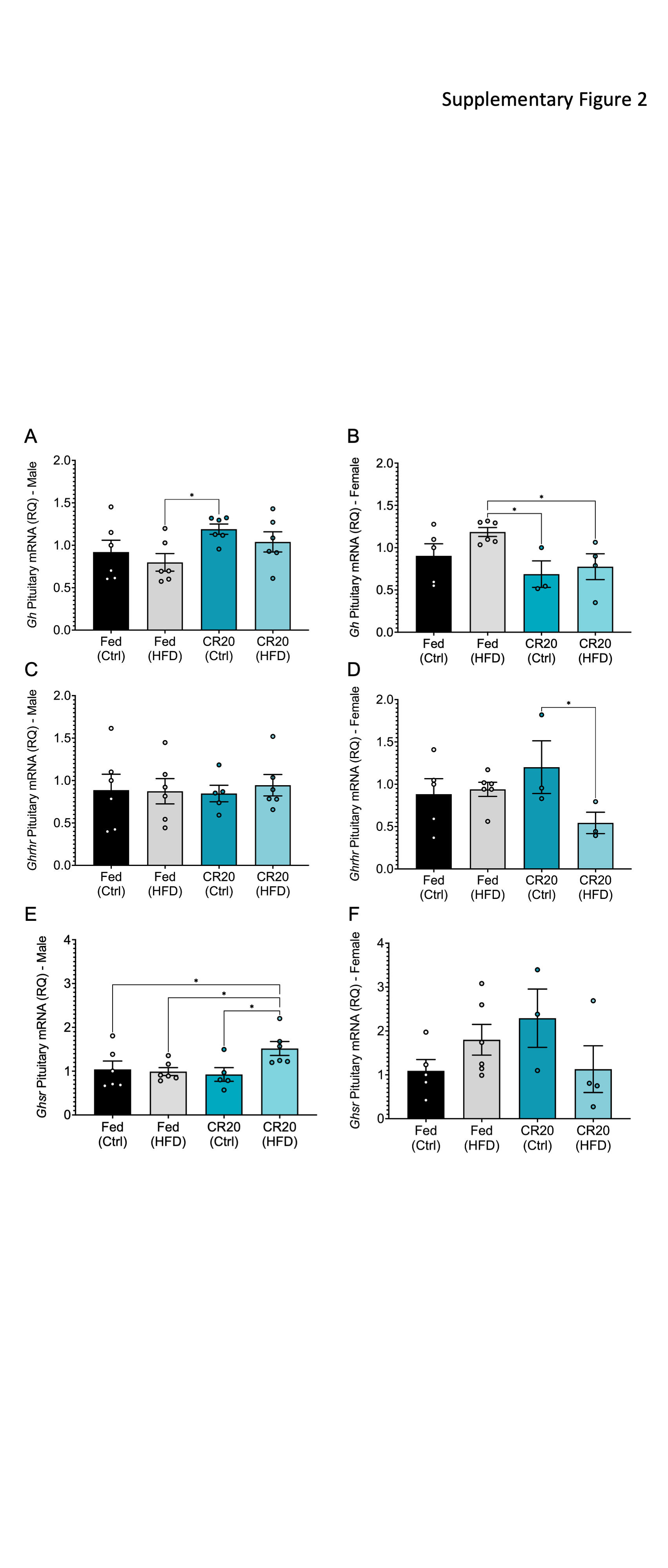

Supplement: Supplementary Figure 1 — Dam correlation analysis of leptin to weight and serum IGF-1 in PND16 pups. (A) Pearson correlation analysis (two-tailed) of weight to leptin for dams revealed a moderate positive correlation for the FED (r=0.6091, p=0.0467) and CR20 (r=0.6794, p=0.0075) groups. (B) Serum IGF1 protein levels in PND16 pups was quantified by ELISA. Student’s t test. [file DataSheet_1.zip › Supplementary Material/Supplementary Figure 2.TIFF]

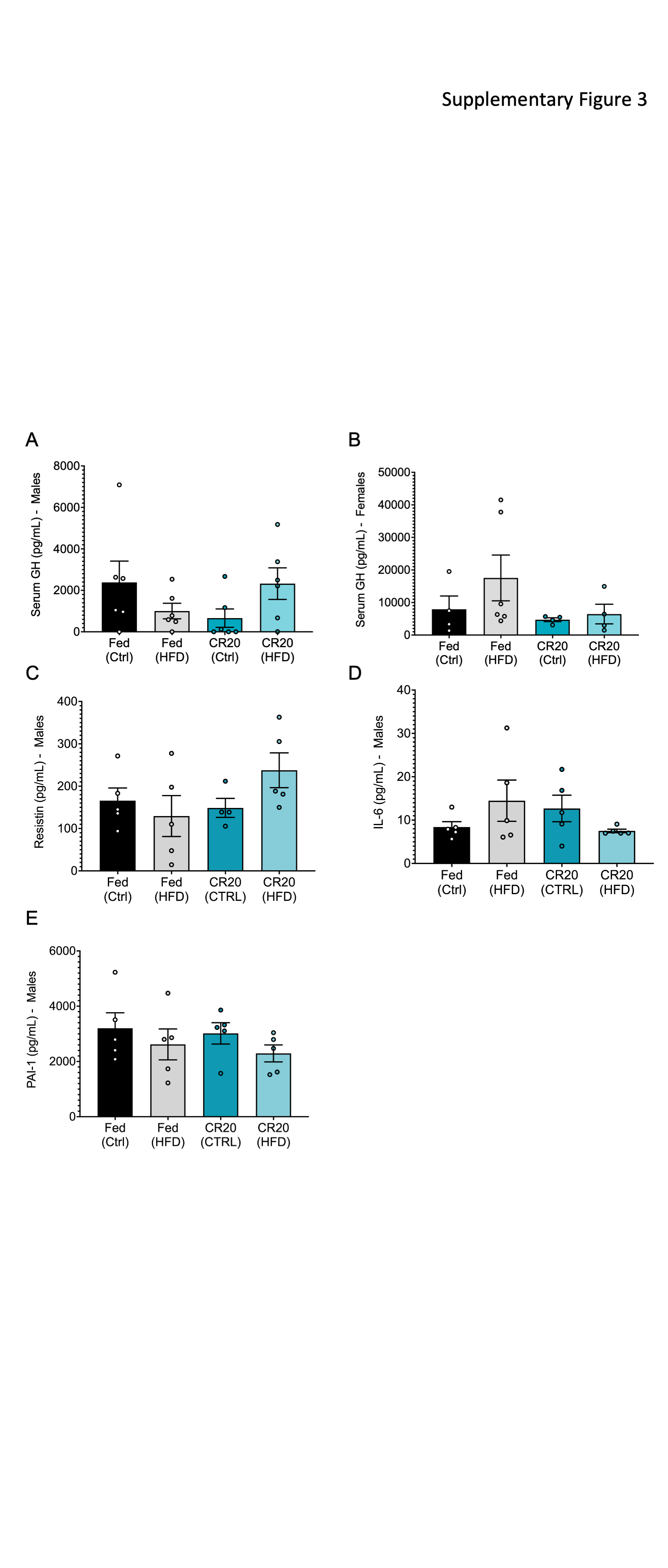

Supplement: Supplementary Figure 1 — Dam correlation analysis of leptin to weight and serum IGF-1 in PND16 pups. (A) Pearson correlation analysis (two-tailed) of weight to leptin for dams revealed a moderate positive correlation for the FED (r=0.6091, p=0.0467) and CR20 (r=0.6794, p=0.0075) groups. (B) Serum IGF1 protein levels in PND16 pups was quantified by ELISA. Student’s t test. [file DataSheet_1.zip › Supplementary Material/Supplementary Figure 3.TIFF]

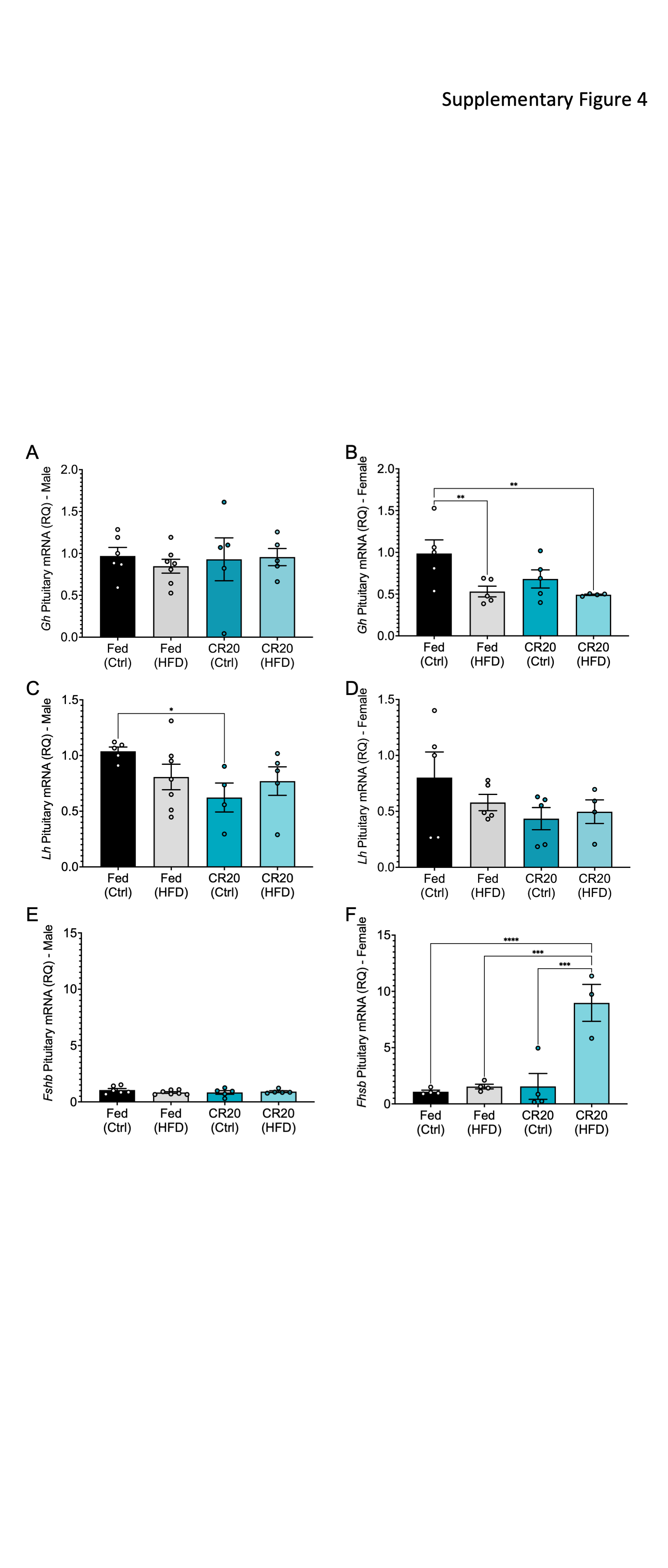

Supplement: Supplementary Figure 1 — Dam correlation analysis of leptin to weight and serum IGF-1 in PND16 pups. (A) Pearson correlation analysis (two-tailed) of weight to leptin for dams revealed a moderate positive correlation for the FED (r=0.6091, p=0.0467) and CR20 (r=0.6794, p=0.0075) groups. (B) Serum IGF1 protein levels in PND16 pups was quantified by ELISA. Student’s t test. [file DataSheet_1.zip › Supplementary Material/Supplementary Figure 4.TIFF]

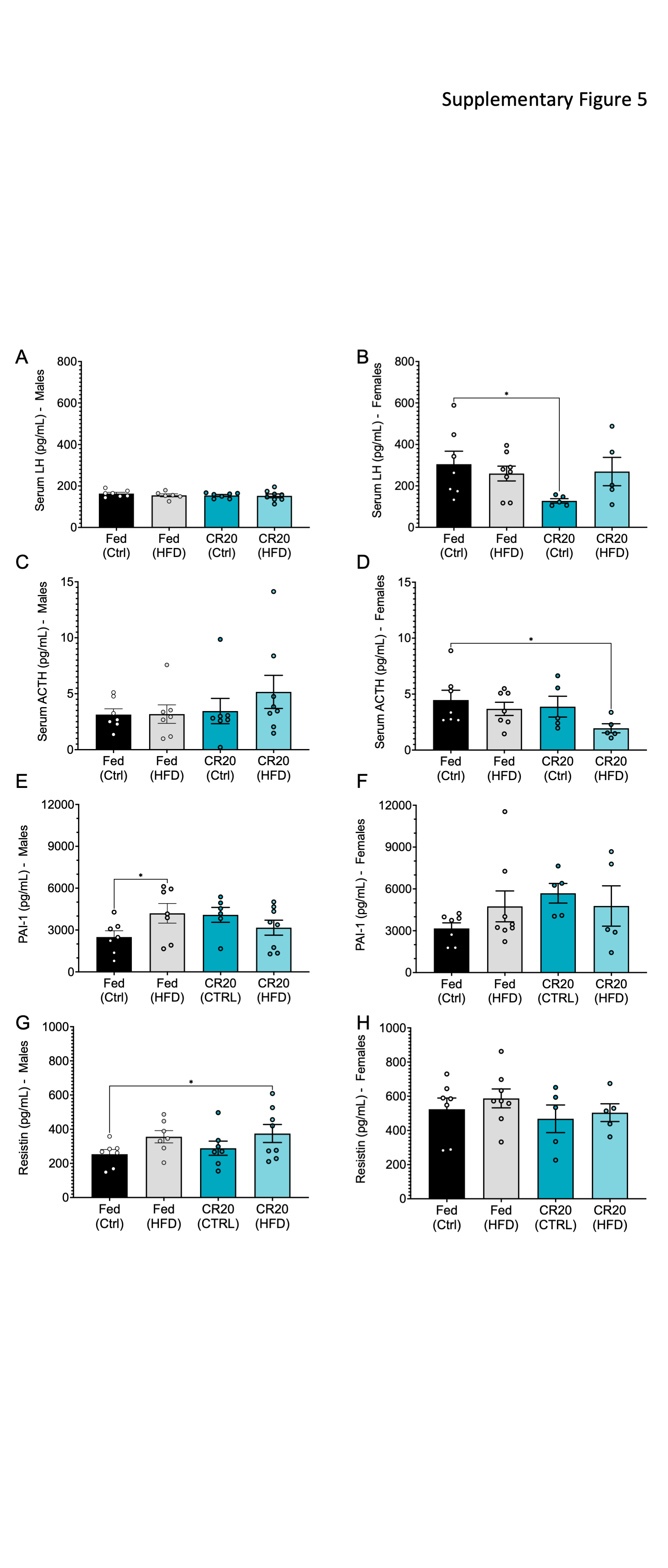

Supplement: Supplementary Figure 1 — Dam correlation analysis of leptin to weight and serum IGF-1 in PND16 pups. (A) Pearson correlation analysis (two-tailed) of weight to leptin for dams revealed a moderate positive correlation for the FED (r=0.6091, p=0.0467) and CR20 (r=0.6794, p=0.0075) groups. (B) Serum IGF1 protein levels in PND16 pups was quantified by ELISA. Student’s t test. [file DataSheet_1.zip › Supplementary Material/Supplementary Figure 5.TIFF]

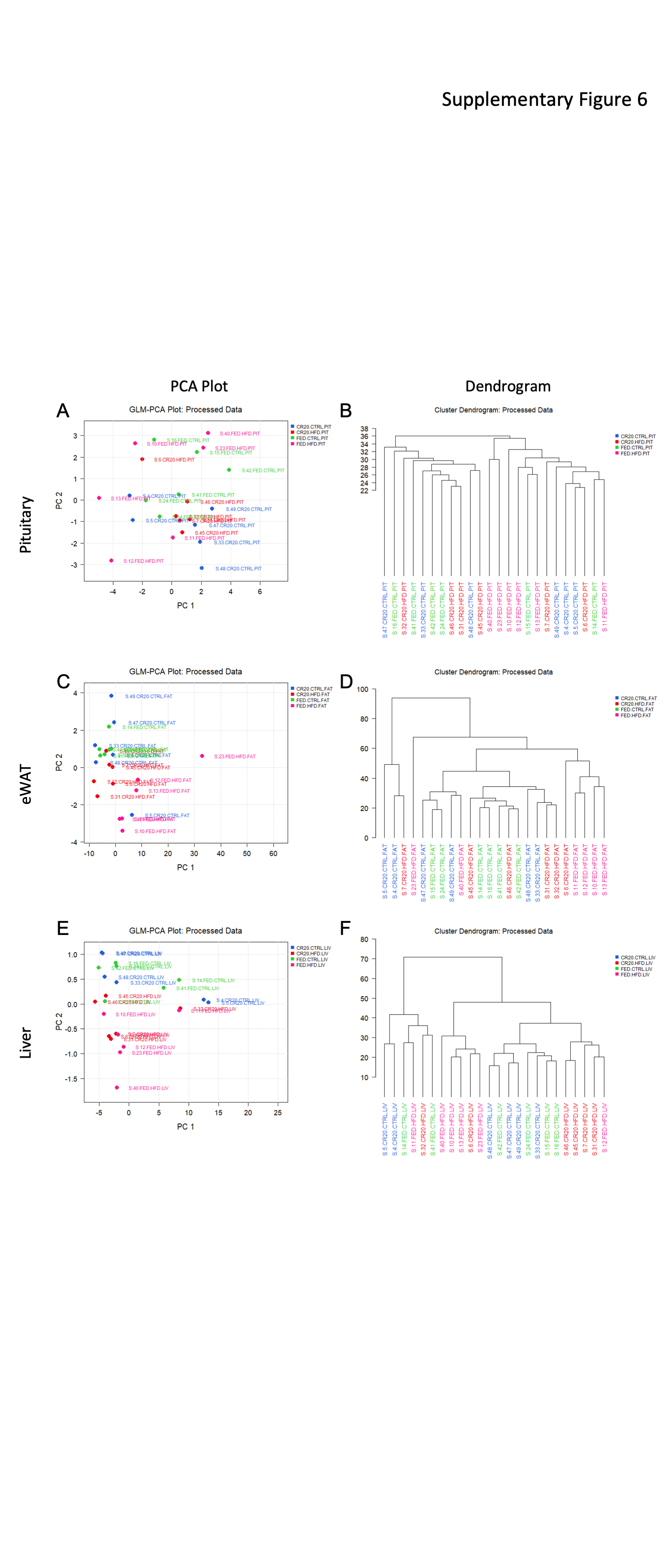

Supplement: Supplementary Figure 1 — Dam correlation analysis of leptin to weight and serum IGF-1 in PND16 pups. (A) Pearson correlation analysis (two-tailed) of weight to leptin for dams revealed a moderate positive correlation for the FED (r=0.6091, p=0.0467) and CR20 (r=0.6794, p=0.0075) groups. (B) Serum IGF1 protein levels in PND16 pups was quantified by ELISA. Student’s t test. [file DataSheet_1.zip › Supplementary Material/Supplementary Figure 6.TIFF]
